# Impaired ability of Nef to counteract SERINC5 is associated with reduced plasma viremia in HIV-infected individuals

**DOI:** 10.1038/s41598-020-76375-w

**Published:** 2020-11-10

**Authors:** Mako Toyoda, Doreen Kamori, Toong Seng Tan, Kageaki Goebuchi, Jun Ohashi, Jonathan Carlson, Ai Kawana-Tachikawa, Hiroyuki Gatanaga, Shinichi Oka, Massimo Pizzato, Takamasa Ueno

**Affiliations:** 1 Joint Research Center for Human Retrovirus Infection, Kumamoto University, 2-2-1 Honjo, Kumamoto, 860-0811 Japan; 2 University of Tokyo, Tokyo, Japan; 3 Microsoft Research, Los Angeles, CA USA; 4 AIDS Research Center, National Institute of Infectious Diseases, Tokyo, Japan; 5 National Center for Global Health and Medicine, Tokyo, Japan; 6 Department of Cellular, Computational and Integrative Biology, University of Trento, Trento, Italy

**Keywords:** Retrovirus, Viral immune evasion, Viral pathogenesis, Virus-host interactions, HIV infections

## Abstract

HIV-1 Nef plays an essential role in enhancing virion infectivity by antagonizing the host restriction molecule SERINC5. Because Nef is highly polymorphic due to the selective forces of host cellular immunity, we hypothesized that certain immune-escape polymorphisms may impair Nef’s ability to antagonize SERINC5 and thereby influence viral fitness in vivo*.* To test this hypothesis, we identified 58 Nef polymorphisms that were overrepresented in HIV-infected patients in Japan sharing the same HLA genotypes. The number of immune-associated Nef polymorphisms was inversely correlated with the plasma viral load. By breaking down the specific HLA allele-associated mutations, we found that a number of the HLA-B*51:01-associated Y120F and Q125H mutations were most significantly associated with a reduced plasma viral load. A series of biochemical experiments showed that the double mutations Y120F/Q125H, but not either single mutation, impaired Nef’s ability to antagonize SERINC5 and was associated with decreasing virion infectivity and viral replication in primary lymphocytes. In contrast, other Nef functions such as CD4, CCR5, CXCR4 and HLA class I downregulation and CD74 upregulation remained unchanged. Taken together, our results suggest that the differential ability of Nef to counteract SERINC5 by naturally occurring immune-associated mutations was associated with the plasma viral load in vivo.

## Introduction

Nef is an accessory protein of HIV-1 and other primate immunodeficiency viruses that is crucial for efficient virus replication in infected individuals and for virus pathogenicity^1,2^. Despite its small size of about 27–35 KDa, Nef performs a striking number of functions, including downregulation of the viral entry receptors (CD4, CCR5 and CXCR4) and HLA class I molecules and upregulation of HLA class II invariant chain (CD74) from the cell surface as well as stimulation of viral replication in CD4^+^ T cells^3–8^. Nef also enhances the infectivity of progeny virions^9–11^ mediated in part by counteracting host serine incorporator (SERINC) 3 and 5, which molecules restrict HIV-1 infectivity, of which SERINC5 is the most potent^12,13^. SERINC5 is incorporated into the membranes of progeny virions in virus-producing cells and antagonizes fusion with target cells^14^. Nef inhibits this process by internalizing SERINC5 from the surface of the virus-producing cells^12^.

Despite being one of HIV-1’s most variable proteins, Nef nevertheless possesses several functionally-important, highly conserved motifs. Motifs responsible for each of Nef’s functions have been identified in mutagenesis studies on laboratory-adapted HIV-1 strains^3–5,15–19^. For instance, the introduction of mutations to the highly conserved FPD motif (Phe121-Pro122-Asp123) in Nef was shown to result in disruption of Nef’s ability to counteract SERINC3/5, thus decreasing the infectivity of progeny virions^12,20^. However, it is unclear whether highly diverse naturally-occurring (patient-derived) Nef sequences also display differential abilities to counteract SERINC3/5; and if so, it remains elusive whether this ability of patient-derived Nef influences viral fitness in vivo. Because Nef is a dominant target of host cellular immunity^21,22^, certain immune-escape polymorphisms might affect the Nef function and plasma viremia. Indeed, recent literature demonstrates that two CTL escape mutations, K94E and H116N, observed in elite controllers impair to some extent Nef’s ability to internalize SERINC5^23^. In the present study, we analyzed HLA-associated Nef polymorphisms inversely associated with the plasma viral load in 375 HIV-infected individuals in Japan and further investigated how these polymorphisms affected Nef’s functions.

## Results

### Nef HLA-associated polymorphisms in chronically HIV-1 subtype B infected subjects

We first sought to identify and characterize HLA-associated Nef polymorphisms in 375 treatment-naïve, chronically HIV-1 subtype B-infected subjects in Japan, a unique population of HLA class I alleles and predominantly HIV-1 subtype B epidemic. A total of 108 HLA class I alleles, defined by four-digit resolution, were observed at frequencies consistent with previous literature^24–26^. Among these, 49 alleles (including 12 HLA-A, 23 HLA-B, and 14 HLA-C) were observed in at least 10 individuals and thus included in the statistical analyses of HLA-associated polymorphisms. HLA-associated polymorphisms were identified by using a phylogenetically corrected logistic-regression model that corrects the potential confounders, which include HLA linkage disequilibrium between host HLA class I alleles, evolutional relationship between the viral sequences, and viral codon covariation^21,27^. The identified HLA-associated amino acid residues in Nef were classified as adapted and non-adapted associations when amino acid enriched or depleted in the presence of a particular HLA^28^. At a threshold of a false-discovery rate (*q* value) of < 0.2, we identified a total of 112 HLA-associated Nef polymorphisms comprising 58 adapted and 54 non-adapted associations occurring at 55 out of 206 codons (Table S1). These numbers were largely consistent with a previously published study showing that a total of 104 HLA-associated Nef polymorphisms occurred at 45 codons in a cohort of an HIV-infected Japanese population (N = 306)^26^. For example, both studies consistently demonstrated that Nef codon 81 was associated with HLA-B*35:01 and HLA-B*39:01, and that Nef codon 135 was associated with HLA-A*24:02, the most prevalent HLA class I allele in this population. However, there were some differences in HLA-Nef codon associations; e.g., 2 Nef polymorphisms at codons 120 and 125 were associated with HLA-B*51:01 in this study, whereas no HLA-B*51:01-associated Nef polymorphism was identified by Chikata et al.^26^.

### Number of Nef HLA-adapted polymorphisms inversely associated with plasma viral load

**Figure 1:**
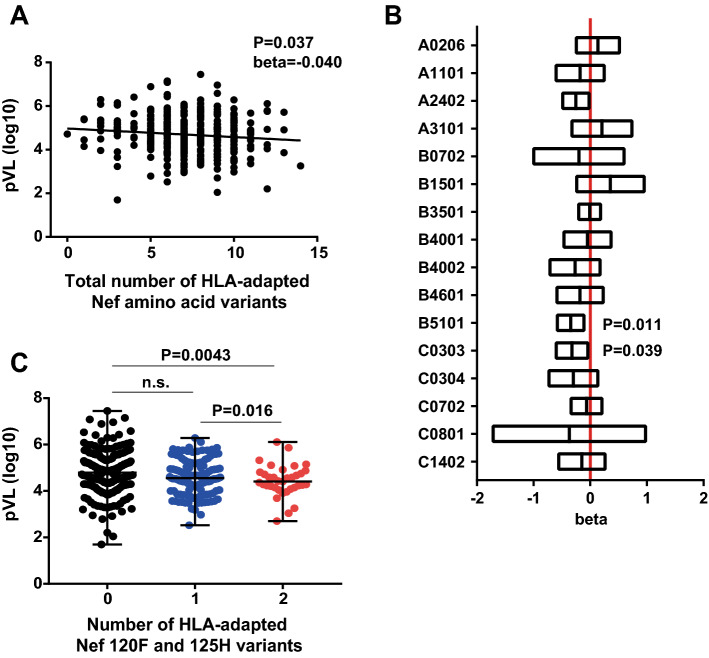
Association between the number of Nef HLA-associated adapted polymorphisms and plasma viral load. (**A**) Results of regression analysis between the total number of HLA-adapted Nef amino acid variants and log10-transformed plasma viral load (pVL) in chronically HIV-1 subtype B infected subjects in Japan (n = 375). (**B**) Regression coefficient and 95% CI obtained from the regression analysis in which pVL and the number of HLA-adapted Nef variants were used as dependent and independent variables for HLA-matched subjects. (**C**) Difference in plasma viral load between the subjects harbouring HLA-adapted Nef 120F and 125H mutations regardless of the HLA class I alleles expressed by the subject. Statistical analysis was performed by using the Mann–Whitney U-test. *n.s.* not significant.

We then sought to investigate the relationship between the presence of HLA-associated amino acid variants in Nef and the plasma viral RNA load (pVL) and CD4 count of the patients. Amino acid variants within a given Nef sequence were counted as HLA-adapted amino acid variants if they had been identified as being HLA-adapted associations in this study, regardless of the HLA class I alleles expressed by the patient. For example, Nef-8C is an HLA-C*15:02-associated adapted polymorphism (Table S1); and, as such, any Nef sequence harboring Cys at codon 8 was counted as having an HLA-adapted amino acid variant at this site. A weak but statistically significant inverse association was observed between pVL and the total number of HLA-adapted Nef amino acid variants (beta =  − 0.040, p = 0.037; Fig. 1A). In contrast, we observed no significant association between the CD4 count and the total number of HLA-adapted Nef amino acid variants (p = 0.10). Although the overall pVL association was weak, these results may raise the interesting hypothesis that selection of certain HLA-driven variants in Nef could modulate viremia in this population.

We postulated that the observed weakness of association between HLA-adapted Nef variants and a lower pVL could be dominantly attributed to only certain HLA-adapted variants. To address this issue, we stratified the HLA-adapted Nef variants associated with 22 prevalent HLA class I alleles (> 10% frequency) in this cohort and tested for association with the pVL values by performing regression analysis; however, only 16 HLA class I alleles were associated with HLA-adapted Nef variants (Fig. 1B). Statistically significant inverse associations were observed between pVL *versus* the number of HLA-B*51:01-adapted variants (beta = − 0.35, p = 0.011) and the number of HLA-C*03:03-adapted variant (beta =  − 0.32, p = 0.039; Fig. 1B). Because there were 2 HLA-B*51:01-adapted variants (120F and 125H) and an HLA-C*03:03-adapted variant (85F) (Table S1), we further examined the association of pVL with the number of the 120F and 125H variants or the number of the 85F variant, regardless of the HLA class I alleles expressed by the subject. Subjects infected with plasma viruses encoding both Nef 120F and 125H mutations exhibited statistically significantly lower pVL compared to those encoding the consensus amino acid residues (Y120 and Q125) or a subset of the mutations (p < 0.02, Mann–Whitney test; Fig. 1C). In contrast, subjects infected with plasma viruses encoding the 85F mutation did not show any significant difference in pVL compared to those encoding the consensus residue (p > 0.05, Mann–Whitney test). One might be concerned that HLA-B*51:01 is a protective allele in this population and that patients expressing either of them had superior immune-mediated viral control; and indeed ^120^YFPDWQNY^125^ has been shown to be a CTL epitope presented by HLA-B*51:01^29,30^. However, no significant association was observed between pVL and this allele (p > 0.05, Mann–Whitney test). Rather, because F121 and D123 are highly conserved residues important for certain Nef functions^31–34^, these results suggest that modulation of pVL may be attributable to altered Nef functions mediated by the HLA-adapted Nef Y120F and Q125H variants.

### Viral replication capacity of HIV-1 Nef variants in primary CD4^+^ cells

**Figure 2:**
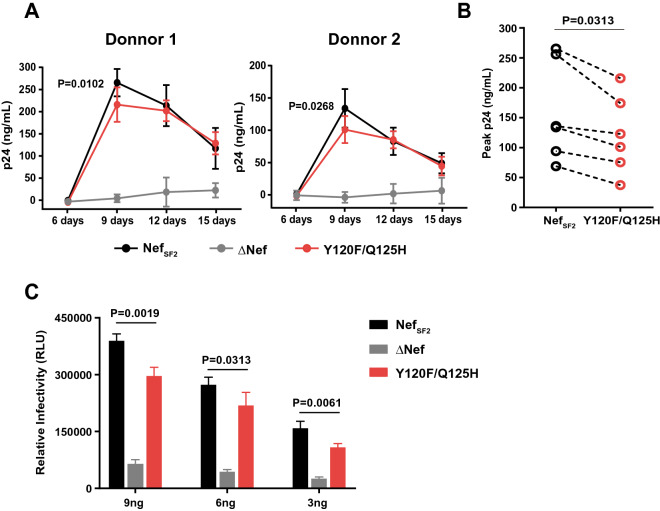
Effects of Nef Y120F/Q125H mutations on viral replication in PBMC. (**A**) PBMC (10^6^ cells) prepared from 2 HIV-negative donors were infected for 6 h with HIV-Nef_SF2_, HIV_ΔNef_ or HIV-Nef_120F/125H_ that had been produced in HEK293T cells at 10 ng of p24 Ag, and then continuously cultured at 37 °C in fresh culture medium for an additional 15 days. Culture supernatants were collected and replaced with fresh medium every 3 days. To monitor viral replication, we quantified the concentration of p24 Ag in the culture supernatant by use of ELISA. HIV-Nef_SF2_, HIV_ΔNef_, and HIV-Nef_120F/125H_ replication kinetics are shown in each panel. P-values were calculated by using the unpaired t-test at Day 9 (HIV-Nef_SF2_ *vs*. HIV-Nef_120F/125H_). (**B**) A series of the same experiments was done by using PBMC prepared from 4 additional HIV-negative donors. The peak p24 Ag values were plotted and statistically analyzed by using the Wilcoxon matched-pairs signed rank test. (**C**) TZM-bl cells (1 × 10^4^ cells) were exposed to HIV-Nef_SF2_, HIV_ΔNef_ or HIV-Nef_120F/125H_ prepared as above at 9, 6, and 3 ng of p24 Ag. Twenty-four hr later, the reporter cells were lysed and β-galactosidase activity generated as a consequence of infection was measured using a chemiluminescence substrate. Data shown are mean ± SD from 3 or 4 independent experiments. P-values were determined by ANOVA with multiple comparisons.

We first tested whether the Y120F and Q125H mutations in Nef impaired viral replication capacity in primary CD4^+^ cells. We prepared PBMC from 2 HIV-negative donors, exposed them to HIV-Nef_SF2_, HIV_∆Nef_ or HIV-Nef_Y120F/Q125H_ at Day 0, and measured time-course changes in p24 Gag antigen secreted into the culture supernatant (as a measure of viral replication) until Day15. As expected, HIV-Nef_SF2_, as compared to HIV_∆Nef_, exhibited a substantial increase in p24 Gag antigen at Day 9 in PBMC from both donors. HIV-Nef_Y120F/Q125H_ exhibited a substantial increase in p24 Gag antigen, but the peak level reached at Day 9 was significantly reduced compared to that of HIV-Nef_SF2_ in PBMC from both donors (p < 0.03; Fig. 2A). We conducted this assay with 4 additional HIV-negative donors. The data demonstrated that the peak level of p24 Gag antigen of HIV-Nef_Y120F/Q125H_ was significantly lower than that of Nef_SF2_ (Wilcoxon matched-pairs test, p = 0.0313; Fig. 2B), indicating that the Nef Y120F and Q125H mutations in combination impaired viral replication.

The observed difference in Nef’s ability to stimulate viral replication could be attributed by viral infectivity of the viral inocula used, because Nef is known to enhance virion infectivity^35^ and the Y120F and Q125H mutations may affect this Nef’s function. Virion infectivity was tested using TZM-bl reporter cells as target cells and the same preparations of viral inocula used in the replication assays. As expected, HIV-Nef_SF2_, as compared to HIV_∆Nef_, exhibited a substantial increase in infectivity, and the level of infectivity was increased proportionally to the amount of the viral inocula used (Fig. 2C). Nef_Y120F/Q125H_ exhibited a substantial increase in infectivity, but ~ 20% reduced level compared to HIV-Nef_SF2_ regardless of the amount of inocula used (p < 0.04; Fig. 2C). These results suggested that the mutations impair Nef’s ability to enhance viral infectivity and stimulate viral replication in primary PBMC.

### Effects of Nef variants on counteraction of SERINC3/5-mediated inhibition of HIV-1 infectivity

**Figure 3:**
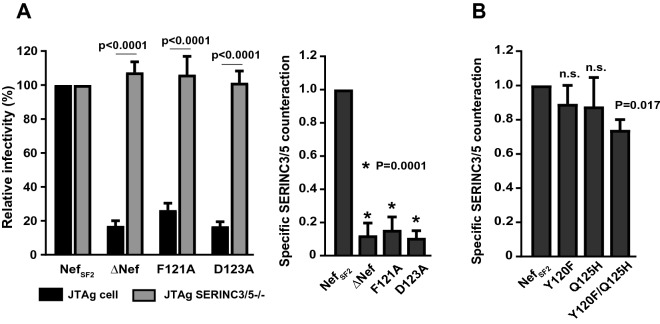
Effects of Nef Y120F and Q125H mutations on counteraction of SERINC3/5-mediated infectivity inhibition. (**A**) Infectivity of viruses that were produced from JTAg cells and JTAg-SERINC3/5^−/−^ cells (*left* panel). Those cells were transduced with HIV-1 NL43 proviral constructs lacking Nef (ΔNef) or carrying Nef_SF2_ and the mutants (F121A and D123A), and then TZM-bl cells were exposed to the resultant viruses to determine viral infectivity. Results are expressed as the mean of triplicate assessments, normalized to the control strain, NL4.3-Nef_SF2_. Statistical analysis was performed by using the paired t test. Ability of Nef_SF2_ and the indicated mutants (F121A and D123A) to counteract SERINC3/5 was determined by dividing the relative infectivity of viral particles secreted from JTAg cells by that from JTAg-SERINC3/5^−/−^ cells (*right* panel). (**B**) Specific SERINC3/5 counteraction function of Nef_SF2_ and the indicated mutants (Y120F, Q125H, Y120F/Q125H). Data shown are the mean results ± SD from 3 independent experiments. Statistical analysis was performed by ANOVA with multiple comparisons *vs* Nef_SF2_. *n.s.* not significant.

SERINC3 and 5 (SERINC3/5) molecules were recently revealed as being inhibitors of HIV-1 virion infectivity and counteracted by HIV-1 Nef^12,13^. So we wanted to examine whether the Y120F and Q125H mutations would impair the ability of Nef to counteract SERINC3/5. Before testing this directly, we first undertook the following control experiments. We transfected JTAg cells with pNL43-Nef_SF2_ and pNL43-∆Nef plasmids, harvested the virus-containing supernatant, and then exposed TZM-bl cells to it. By measuring the luminescence intensity generated from HIV-infected TZM-bl cells, we assessed the virion infectivity. Relative infectivity was calculated as luminescence intensity obtained by HIV-Nef_SF2_ normalized to 100%. As expected, infectivity of HIV-Nef_SF2_ was much enhanced as compared to that of HIV_∆Nef_ (Fig. 3A). Moreover, when we transfected JTAg cells that had been engineered to knock out both SERINC3/5 (JTAg-SERINC3/5^−/−^) with the same pNL43-Nef_SF2_ and pNL43-∆Nef plasmids, both virus preparations showed comparable infectivity (Fig. 3A), clearly confirming that SERINC3/5 were inhibitors of virion infectivity counteracted by Nef. We defined the specific ability of Nef to counteract SERINC3/5 by calculating infectivity of viral particles secreted from parental JTAg cells divided by that of JTAg-SERINC3/5^−/−^ cells, such that values > 1.0 and < 1.0 indicated increased or decreased ability of Nef to counteract SERINC3/5 compared to Nef_SF2_, respectively (Fig. 3A). Testing of the F121A and D123A mutations in Nef_SF2_ showed nearly complete disruption of Nef’s ability to counteract SERINC3/5 (Fig. 3A), confirming previously reported findings^12^. We then tested the Nef_SF2_ harboring the Y120F, Q125H, and Y120F/Q125H mutations for SERINC3/5 counteraction. No substantial effects were observed with the single mutations; whereas the double Y120F/Q125H mutation showed ~ 20% reduced ability to counteract SERINC3/5 (Fig. 3B).

### Functional effects of Nef variants in the context of patient-derived Nef sequences

**Figure 4:**
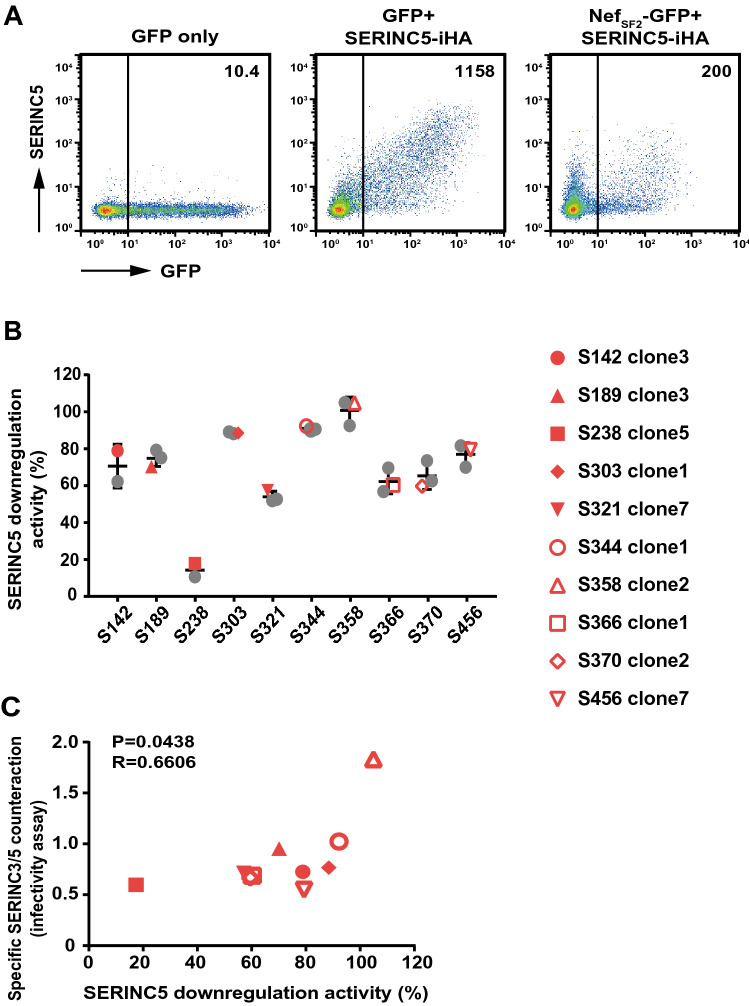
Effects of patient-derived Nef clones on SERINC5 downregulation and counteraction of SERINC3/5-mediated infectivity inhibition. (**A**) Representative flow cytometry plots display the expression of surface SERINC5 in JTAg-SERINC3/5^−/−^ cells that had been co-transfected with genes encoding GFP and Nef _SF2_-GFP together with SERINC5-iHA or an empty vector. The resultant cells were analyzed for cell-surface SERINC5 expression (as HA) and GFP. MFI values for SERINC5-iHA in the GFP^+^ subset are shown. (**B**) Nef clones isolated from the indicated HIV-infected subjects (2 or 3 clones per patient) were analyzed for their ability to downregulate SERINC5-iHA. Results are expressed as relative downregulation activity, normalized to the control strain, Nef_SF2_. Plots indicated in red were used for functional analyses in panel C and Fig. 5. (**C**) Correlation analysis between Nef’s ability to counteract SERINC3/5 (for viral infectivity) and to downregulate cell surface expression of SERINC5. Nef clones tested are indicated in (**B**). Statistical analysis was done with the Spearman test.

Mutational effects on Nef functionality are often dependent on backbone sequences or genetic lineages. We first analyzed the sequence of amplified *nef* gene fragments after they had been cloned into a plasmid (average of 8 clones per subject) in 10 out of 12 HLA-B*51:01^+^ patients whose autologous viruses had the Y120F/Q125H mutations. Nef clones clustered closely with their respective bulk plasma HIV RNA sequences in the phylogenetic tree (Fig. S1A). In 6 patients, all sequenced clones had both Y120F/Q125H mutations; whereas in the other patients, a minority of the Nef clones had the single Y120F mutation (Fig. S1B). Next, by the use of a transfection-based assay, 2 or 3 Nef sequences harboring the Y120F/Q125H mutations were tested for Nef’s ability to downregulate SERINC5 from the cell surface. SERINC5-iHA expression was increased when JTAg-SERINC3/5^−/−^ cells were transfected with DNA encoding SERINC5-iHA, whereas co-transfection with DNA encoding SERINC5-iHA and Nef_SF2_-GFP resulted in substantial reduction in cell surface expression of SERINC5-iHA (Fig. 4A), confirming Nef’s ability to downregulate SERINC5^23,36,37^. All patient-derived Nef clones tested were functional with respect to the SERINC5-iHA downregulation function, but the activity level was different to a relatively small extent within a host, but to a large extent across hosts (Fig. 4B). A *nef* clone from each patient (shown by the red plots in Fig. 4B and their amino acid sequences are given in Fig. S2) was subcloned into pNL43, and infectivity potential of the recombinant viruses harboring patient-derived Nef clones was determined. We observed a weak but statistically significant correlation in Nef functions between SERINC5-iHA downregulation and the specific counteraction of SERINC3/5 in the infectivity assay (Spearman R = 0.6606, p = 0.0438; Fig. 4C).

**Figure 5:**
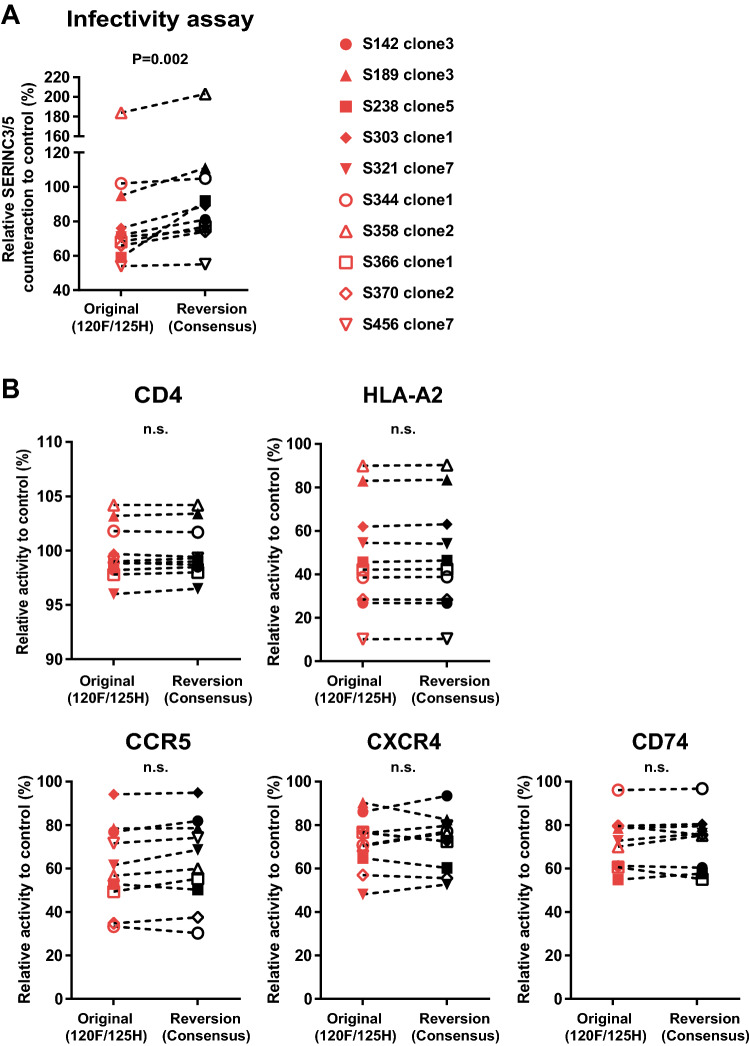
Effects of Y120F and Q125H mutations in patient-derived Nef clones on counteraction of SERINC3/5-mediated infectivity inhibition and modulation of cell surface receptor expression. Patient-derived Nef clones (n = 10) harboring HLA-B*51:01-adapted 120F and 125H mutations (see legend to Fig. 4B) were subcloned into HIV-1 pNL43 or Nef-GFP fusion plasmids, and their corresponding Y120 and Q125 reversion mutants were constructed. Pair-wise analyses of Nef’s ability to counteract SERINC3/5 (**A**) as well as downregulate CD4, CCR5, CXCR4 and HLA class I, and upregulate CD74 (**B**) were conducted. Data represent indicated Nef functions by parental and revertant clones. Data shown are the mean results ± SD from 3 or 4 independent experiments. Statistical analysis was performed by the Wilcoxon matched-pairs signed rank test. *n.s.* not significant.

Finally, to validate the effects of the Y120F/Q125H mutations on Nef function in the context of patient-derived Nef sequences, we reverted each Nef clone to the consensus Y120 and Q125 to generate paired mutant/revertant constructs. The pair-wise comparison showed that Nef’s ability to counteract SERINC3/5 was restored when the reversions, from 120F/125H to Y120/Q125, were introduced (Wilcoxon matched-paired test, P = 0.002; Fig. 5A). In contrast, Nef’s ability to downregulate CD4, CCR5, CXCR4 and HLA-A*02 and upregulate CD74 was not influenced by the reversions (Fig. 5B). These results indicate that the HLA-B*51:01-adapted Y120F/Q125H mutations selectively impaired Nef’s ability to counteract SERINC3/5 but that CD4, CCR5, CXCR4 and HLA downregulation functions and CD74 upregulation function remained unaffected.

## Discussion

We have demonstrated here that a number of naturally-occurring Nef variants (Y120F and Q125H) associated with a certain HLA class I allele (HLA-B*51:01) inversely correlated with the plasma viral load in treatment-naïve HIV-infected patients harboring this HLA allele. In addition, a set of in vitro functional analyses of the Nef variants demonstrated that these 2 mutations in combination selectively impaired Nef’s ability to antagonize the restriction function of SERINC5, associating with decreased viral replication and virion infectivity while preserving other Nef functions including downregulation of HLA class I, CD4, CCR5, and CXCR4 as well as upregulation of CD74. Our findings indicate that certain HLA-associated Nef variants were associated with a decreased plasma viral load and impaired Nef’s ability to antagonize SERINC5 function.

It has been well documented that, by performing mutational analyses, highly conserved Nef residues, including PxxP72, D123, and DD174, 175 are responsible for Nef’s important functions such as HLA downregulation, dimerization, and CD4 downregulation, respectively^19,33,38–41^. In addition, analyses of patient-derived Nef sequences have revealed the contribution of naturally-occurring polymorphisms at Nef’s more variable sites on Nef functions of patient-derived sequences^42–47^. In our study, the immune-escape mutations, Y120F, Q125H, and the double mutations (Y120F/Q125H), exhibited the prevalence of 39.2, 13.1, and 9.3%, respectively; in this study cohort in Japan, the values are much higher than those of subtype B sequences in Los Alamos Database, which are 13.6, 5.2, and 1.6%, respectively. This suggests a unique population of HLA class I alleles of this cohort in Japan, compared to the regions with subtype B epidemic; and implicates that the double mutations could not be prevalent without HLA-driven selective pressure. However, the double mutations are relatively prevalent in subtype D (11.9%) in the data from Los Alamos database, compared to subtype A and C (3.9 and 1.3%, respectively). Further detailed studies are needed to clarify the effects of the Y120F and Q125H mutations on Nef functions of different subtype backbones.

The highly conserved D123 residue (> 99% prevalence in subtype A, B, C and D) is encompassed by the immune-escape mutation sites of Y120F and Q125H. Several studies demonstrate that introduction of mutations at this residue results in impairment of multiple Nef functions including downregulation of CD4 and HLA as well as enhancement of viral infectivity and replication^31–33,48,49^. In addition, D123A mutant impairs Nef’s ability to counteract SERINC3/5 as shown in this study. Crystal structural analysis of the Nef core domain demonstrates that D123 involves a dimerization interface toward R105^48^. Indeed, D123N mutation prevents Nef core dimer formation when bound to Hck SH3 of Src-family kinases^50^. It is thus interesting to see whether and to what extent the immune-escape mutations at the neighboring residues of Y120F and Q125H play a secondary role in Nef dimerization.

Some limitations of our study merit mention. Although we investigated Nef clones isolated from 375 treatment-naïve HIV-infected patients in Japan, this panel did not capture the entirety of subtype B Nef genetic diversity. Also, the cohort of 375 individuals did not capture the entirety of host genetic diversity at HLA class I loci in this population. Several key Nef functions, including enhancing viral replication and virion infectivity, as well as downregulation of SERINC5, CD4, CCR5, CXCR4, and HLA class I molecules and upregulation of CD74, were tested in in vitro assays using Nef variants harboring HLA-associated mutations; but we could not rule out a role for other known or unknown Nef functions. For instance, SERINC3 is also known to restrict HIV-1 infectivity^12,13,51,52^, albeit much less extent compared to SERINC5, and counteracted by HIV-1 Nef. The Nef mutations tested here may differently affect the counteraction functions against SERINC3 and 5. This issue was not specifically addressed here. Due to the limited availability of PBMC samples from the HIV-infected donors, HLA class I-restricted immune responses to Nef variants could not be experimentally addressed. Also, because of the cross-sectional sampling of patients’ specimens in this study, intra-host changes of *nef* sequences over time could not be tested. Despite these limitations, our study provides strong evidence that naturally-occurring variations in Nef-mediated SERINC5 counteraction function may contribute, at least to some extent, to clinical outcomes in HIV-1 infections. Our results highlight the conflicting fitness effects of Nef arising by the interplay between antiviral immunity and intrinsic restriction by the host.

## Materials and methods

### Study subjects

Plasma samples were collected from 446 HIV-1 subtype B chronically infected and treatment-naïve patients who were monitored at the National Center for Global Health and Medicine, Tokyo and Institute of Medical Science, University of Tokyo, Japan from 1996 to 2012. The HLA class I typing of these patients was done by using a high-resolution sequence-based typing protocol as previously described^25^. In a subset of the cohort (N = 375), the data for CD4 counts (median: 307 [IQR: 190.3 to 400.5]/mm^3^) and plasma viral load (median: 45,000 [IQR: 16,000 to 165,000] copies/ml) were available. This study was approved by the Human Research Ethics Committee of the National Center for Global Health and Medicine and the Institutional Review Board of the University of Tokyo, and was conducted at Joint Research Center for Human Retrovirus Infection, Kumamoto University, Kumamoto, Japan, according to the principles expressed in the Declaration of Helsinki. Written informed consent was obtained from all study participants.

### Sequence analysis of autologous nef genes

After precipitation of HIV-1 particles by ultracentrifugation (50,000 rpm, 15 min) of patients’ plasma, the viral RNA was purified by use of a QIAamp Viral RNA Min kit (Qiagen) followed by the synthesis of cDNA carried out with a Cloned AMV First-strand cDNA Synthesis Kit (Invitrogen Corp, Carlsbad, CA), as previously describe^24^. Through nested PCR, DNA fragments spanning the *nef* gene were amplified with the set of primers previously described^53^. The resultant PCR product was purified and directly sequenced with an automated sequencer (ABI 3500/3500XL; Applied Biosystems, Carlsbad, CA). The sequence data were analyzed and aligned by using Seqscape software version 2.7 and Gene cutter tool in the Los Alamos sequence database (https://www.hiv.lanl.gov/) with respect to a reference HXB2 strain. To facilitate a consistent codon numbering scheme (based on the Nef_HXB2_ reference strain), we pairwise-aligned all *nef* sequences to *nef*_HXB2_ by using an in-house algorithm based on the HyPhy platform^54^ and insertions stripped out.

### Cloning and plasmid construction

Patient-derived *nef* genes amplified as above were cloned into a plasmid by using a Zero Blunt TOPO PCR Cloning kit (Invitrogen). A median of 8 *nef* clones was sequenced per patient. To examine functionality of *nef* gene products, control (strain SF2) and the patient-derived Nef sequences were subcloned in the pcDNA3.1-GFP plasmid^55^ and HIV-1_NL43_ proviral construct as previously described^43,56,57^. Defined mutations of interest were then introduced by using overlapping PCR^22,58^. All control, patient-derived and mutation-introduced plasmid constructs were re-confirmed by DNA sequencing of the entire *nef* region.

### Analysis of Nef-mediated modulation of cell surface molecules

Nef-mediated downregulation of cell surface CD4 and HLA-I molecules was assessed in CEM, a human CD4^+^ T cell line stably transfected with the gene encoding HLA-A*02:01 (CEM-A*02 cells). CEM-A*02 cells (2 × 10^6^) were electroporated with 8 μg plasmid DNAs encoding GFP alone or Nef-GFP fusion proteins by electroporation in 0.4-mm cuvettes under the following Gene Pulser Xcell square-wave conditions: 250 V, 25 ms, and 1 pulse (Bio-Rad Laboratories, Inc.). Twenty-four hour later, the resultant cells were stained with brilliant violet-conjugated anti-human CD4 mAb and HLA-A2 serotype-specific mAb and 7-amino-actinomycin D (7-AAD) (all from BioLegend), as previously described^46,47,57,59^. Nef-mediated downregulation of cell surface CCR5 and CXCR4 molecules was assessed in TZM-bl cells. TZM-bl cells (1 × 10^5^) were transfected with plasmid DNAs encoding GFP alone or Nef-GFP fusion proteins by Lipofectamine 2000 (Themo Fisher). Forty-eight hour later, the resultant cells were stained with phycoerythrin-conjugated anti-human CCR5 mAb, allophycocyanin-conjugated anti-human CXCR4 mAb (BioLegend), and 7-AAD, as previously described^46^. Nef-mediated upregulation of cell surface CD74 molecule was assessed in 721.221 cells. 721.221 cells (1 × 10^5^) were exposed with the recombinant viruses harboring the patient-derived Nef sequences of interest. Forty-eight hour later, the resultant cells were stained with phycoerythrin-conjugated anti-CD74 (BioLegend), 7-AAD, and anti-p24 Gag-FITC (Beckman Coulter), as described^57^. Nef-mediated internalization of SERINC5 from the cell surface was assessed in JTAg cells with SERINC3/5 knocked out (denoted as JTAg-SERINC3/5^−/−^)^12^. JTAg-SERINC3/5^−/−^ cells (3 × 10^6^) were electroporated with 5 μg of pBJ5-SERINC5-internal HA tag (S5-iHA; kindly provided by H. Gottlinger)^13^ together with 5 μg of DNAs encoding GFP alone or Nef-GFP fusion proteins by using the Gene Pulser Xcell square-wave conditions described above. Twenty-four hr later, the resultant cells were stained with Alexa Fluor 647 anti-HA.11 and Zombie Aqua to remove dead cell fractions (all from BioLegend)^36^.

Note that in all systems described above, live cells were gated, and the mean fluorescence intensity (MFI) of CD4, CCR5, CXCR4, HLA-I, or CD74 in Nef-expressing cells (defined as the GFP^+^ or p24 Gag^+^ subset in the transduced cells, denoted MFI Nef^+^ in the below calculation) and non Nef-expressing cells (defined as the GFP^−^ or p24 Gag^−^ subsets in the transduced cells, denoted MFI Nef^−^) was analyzed by flow cytometry (FACS Verse: BD Biosciences). The following formula was used to calculate the CD4, CCR5, CXCR4 and HLA-I downregulation activity and CD74 upregulation activity of each Nef clone: (MFI Nef^−^ − MFI Nef^+^)/MFI Nef^−^ × 100. The MFI value of SERINC5-iHA downregulation activity for each Nef clone was normalized to the negative (GFP^+^/S5-iHA^+^) and positive (Nef_SF2_-GFP^+^/S5-iHA^+^) controls by using the following formula: (MFI_negative_ − MFI_clone_)/(MFI_negative_ − MFI_positive_) × 100. All Nef functional values were reported as the mean of a minimum of triplicate experiments.

### Infectivity assay for SERINC3/5 activity

The recombinant viruses were produced by electroporation of JTAg or JTAg-SERINC3/5^−/−^ cells with NL43-based proviral clones lacking or harboring various *nef* sequences. Twenty-four hour later, the virus-containing supernatant was harvested and quantified by assessing reverse transcriptase activity by using a one-step SYBR green I-based product-enhanced reverse transcriptase assay as described earlier^60,61^. TZM-bl reporter cells (NIH AIDS Research and Reference Reagent Program) were seeded into 96-well plates, exposed to the viruses for 24 h, and then lysed for measurement of β-galactosidase activity by using a Galacto-Star Reporter Assay System (Applied Biosystems) as described previously^57^. To obtain relative infectivity of the viruses, we divided the number of infected cells (as measured by luminescence value) by the amount of the input virus (as measured by reverse transcriptase activity), and then normalized it to the control strain, NL4.3-Nef_SF2_. Furthermore, for quantification of Nef’s ability to counteract SERINC3/5, the relative infectivity value of the JTAg cell-derived virus was divided by that of the JTAg-SERINC3/5^−/−^ cell-derived virus. Thereby, values > 1.0 and < 1.0 respectively indicated increased and decreased ability of Nef to counteract SERINC3/5. All Nef functional values were reported as the mean of a minimum of triplicate experiments.

### Viral replication assay

HEK293T cells seeded on 6-well plates were transfected with NL43-based proviral clones harboring various *nef* sequences. Forty-eight hour later, virus-containing supernatants were harvested, quantified for the amount of p24^Gag^ Ag by use of ELISA (ZeptoMetrix Corp.) and stored at − 80 °C until use. Freshly isolated PBMC from HIV-negative donors (10^6^ cells) were exposed to the virus preparations (10 ng of p24^Gag^ Ag) for 6 h, washed twice, and resuspended in culture medium (RPMI 1640, 10% fetal calf serum) as described previously^22,57^. Three days later, the PBMCs were stimulated with PHA. Culture supernatants were collected and replaced with fresh medium supplemented with human rIL-2 every 3 days. Viral replication was monitored by measuring p24^Gag^ Ag in the culture supernatant by using ELISA over a 15-day period. ELISA values during the initial burst of viral replication (on day 9) were used as our measure of replication capacity. Results were expressed as the mean of quadruplicate assessments of each donor, normalized to control strain NL4.3-Nef_SF2_.

### Statistical analysis

#### Association between viral polymorphisms and host HLA class I alleles

The published phylogenetic dependency network model (PDN) was used to determine viral polymorphisms that were statistically associated with host HLA class I alleles with a pre-defined threshold of p < 0.05, q < 0.2, as previously described^21,27,62,63^. The PDN model is a phylogenetically corrected logistic regression model that corrects the potential confounders, which include HLA linkage disequilibrium between host HLA class I alleles, evolutional relationship between the viral sequences, and viral codon covariation^21,27,62,63^. The HLA-associated amino acid residues in Nef protein were classified as adapted and non-adapted associations when amino acid enriched or depleted in the presence of a particular HLA^21,28^.

#### Association between viral polymorphisms and clinical parameters

The association of the number of HLA-adapted Nef amino acid variants with log10-transformed pVL or CD4 count was assessed by regression analysis. A two-tailed p-value < 0.05 was considered to be statistically significant.

#### Accession numbers

GenBank accession numbers for clonal nef sequences of subtypes B in this study are LC547123 to LC54720.

## Supplementary information

Supplementary Information.
