# Supplementary material for: Impaired ability of Nef to counteract SERINC5 is associated with reduced plasma viremia in HIV-infected individuals
Source: Sci Rep. 2020 Nov 10;10:19416. doi: 10.1038/s41598-020-76375-w (PMC7656250; doi:10.1038/s41598-020-76375-w)
Supplement: Supplementary file 1 — Supplementary Information. [file 41598_2020_76375_MOESM1_ESM.pdf]

Supplementary materials (Table S1 and Figure S1, S2)

Impaired ability of Nef to counteract SERINC5 is associated with reduced plasma viremia in HIV-infected individuals

Mako Toyoda[1*], Doreen Kamori[1*], Toong Seng Tan[1], Kageaki Goebuchi[1], Jun Ohashi[2], Jonathan Carlson[3], Ai Kawana-Tachikawa[4], Hiroyuki Gatanaga[1,5], Shinichi Oka[1,5], Massimo Pizzato[6], Takamasa Ueno[1#]

[1]Joint Research Center for Human Retrovirus Infection, Kumamoto University, Japan; [2]University of Tokyo, Tokyo, Japan; [3]Microsoft Research, Los Angeles, CA, USA; [4]AIDS Research Center, National Institute of Infectious Diseases, Tokyo, Japan; [5]National Center for Global Health and Medicine, Tokyo, Japan; [6]Department of Cellular, Computational and Integrative Biology, University of Trento, Italy

[*]MT and DK contributed equally to this work.
[#]Corresponding author; uenotaka@kumamoto-u.ac.jp

Table S1 HIV-1 Nef HLA-associated polymorphisms in in the Japanese cohort (q < 0.2).

The direction of the association (adapted vs. nonadapted) for the specific HLA allele in question is specified. P-values and q-values represent the minimum values observed using a phylogenetically corrected logistic regression model. The columns TT-FF denote the 2x2 contingency table. TT=number of patient with both HLA allele and the target amino acid. TF=number of patients with HLA allele and without the target amino acid.

Predictor HLA	Target Position	Target AA	onsensus A	Direction	TT	TF	FT	FF	N	P Value	Q Value
C1502	8	C	S	Adapted	2	14	1	249	266	0.00065	0.194246
A2603	9	G	S	Adapted	2	8	9	208	227	0.00046	0.157065
C1402	10	A	I	NonAdapted	0	32	8	242	282	0.00048	0.174964
A3101	15	D	A	Adapted	6	52	6	298	362	0.00021	0.105549
B3901	15	A	A	NonAdapted	8	15	214	126	363	9.03E-05	0.062247
B3901	23	A	A	NonAdapted	4	17	201	131	353	0.00026	0.114549
B1301	28	D	E	NonAdapted	0	9	133	237	379	0.00025	0.114117
B5901	33	V	V	NonAdapted	5	12	163	160	340	0.00026	0.114549
C0602	39	R	R	NonAdapted	0	12	163	176	351	0.00011	0.071486
B1511	43	V	I	Adapted	2	11	9	358	380	0.00035	0.132371
B5502	45	I	S	Adapted	2	14	4	360	380	0.00021	0.105549
C0803	47	N	N	NonAdapted	9	2	335	11	357	0.00036	0.135227
C0801	49	P	A	Adapted	3	22	4	192	221	0.0004	0.144775
C0701	50	T	A	Adapted	4	2	74	291	371	0.00044	0.174964
C1502	50	S	A	Adapted	2	20	21	328	371	0.0006	0.197341
B4001	55	S	C	Adapted	4	35	4	315	358	0.00046	0.157065
C0702	56	T	A	Adapted	4	109	5	262	380	0.00029	0.121041
B4006	58	V	L	Adapted	5	17	13	247	282	0.00024	0.114549
B1301	60	S	A	Adapted	2	7	4	344	357	0.00031	0.123216
A1101	63	E	E	NonAdapted	25	35	172	126	358	0.00027	0.114549
B3501	63	E	E	NonAdapted	14	30	183	131	358	0.00032	0.12772
B3501	63	D	E	Adapted	30	14	120	190	354	0.00051	0.167189
C0102	64	D	E	NonAdapted	0	134	14	232	380	0.00034	0.130147
B6701	64	E	E	NonAdapted	11	3	312	32	358	0.00052	0.167189
B4006	65	E	E	NonAdapted	4	26	310	41	381	1.78E-19	1.47E-15
B4006	65	D	E	Adapted	20	10	24	327	381	1.40E-14	5.79E-11
C0702	71	R	R	NonAdapted	62	53	249	18	382	2.18E-18	1.35E-14
C0702	71	K	R	Adapted	44	71	16	251	382	2.06E-15	1.02E-11
B0702	71	T	R	Adapted	9	40	5	328	382	4.22E-06	0.004909
B5502	71	K	R	Adapted	6	10	54	312	382	0.00041	0.146607
C0702	80	T	T	NonAdapted	100	15	262	5	382	2.01E-05	0.019203
C0702	80	N	T	Adapted	9	106	2	265	382	0.00031	0.123216
B3501	81	Y	Y	NonAdapted	13	37	244	88	382	1.17E-12	4.16E-09
B3501	81	F	Y	Adapted	37	13	81	251	382	3.77E-12	1.17E-08
B3901	81	F	Y	Adapted	17	8	101	254	380	9.82E-07	0.001807
B3901	81	Y	Y	NonAdapted	6	16	234	97	353	5.99E-05	0.051261
B6701	81	F	Y	NonAdapted	0	15	118	247	380	0.00038	0.142522
C0102	82	K	K	NonAdapted	117	11	226	6	360	6.77E-05	0.054206
B4003	82	Q	K	Adapted	2	2	3	375	382	0.00021	0.102941
B1501	83	G	G	Adapted	37	6	215	96	354	3.26E-05	0.029946
B5201	83	A	G	NonAdapted	9	68	73	209	359	8.88E-05	0.066039
A1101	83	A	G	NonAdapted	7	53	75	224	359	0.00051	0.167189
B5101	83	G	G	NonAdapted	29	27	235	91	382	0.00039	0.160428
C1402	83	A	G	Adapted	19	24	74	265	382	0.00047	0.174964
A0206	85	L	L	NonAdapted	11	50	146	147	354	6.54E-09	1.80E-05
A0206	85	F	L	Adapted	32	29	97	196	354	3.46E-06	0.004312
C0304	85	V	L	NonAdapted	2	78	62	240	382	6.64E-06	0.007171
A1101	85	V	L	NonAdapted	2	61	62	257	382	4.56E-05	0.040417
C0303	85	F	L	Adapted	45	39	94	204	382	9.40E-05	0.06247
B0702	88	S	S	NonAdapted	42	3	299	10	354	0.00059	0.179585
A1101	92	K	K	NonAdapted	37	23	262	38	360	2.11E-07	0.000475
A1101	92	R	K	Adapted	22	38	38	262	360	1.14E-06	0.001886
B4003	93	D	E	Adapted	4	0	24	332	360	1.32E-06	0.002046
B4003	93	E	E	NonAdapted	0	4	328	28	360	3.28E-06	0.004525
B4403	93	E	E	NonAdapted	23	11	305	21	360	0.00026	0.116349
B4001	98	E	E	NonAdapted	16	23	239	82	360	4.21E-07	0.00087
B4001	98	D	E	Adapted	22	17	81	240	360	1.73E-06	0.002531
B4002	98	D	E	Adapted	23	30	78	227	358	0.00012	0.071486
B4002	98	E	E	NonAdapted	30	23	225	80	358	0.00016	0.088169
A0101	99	G	G	NonAdapted	9	2	347	2	360	0.00058	0.178568
A3101	101	T	I	Adapted	4	53	1	298	356	0.00017	0.089266
C0304	101	I	I	Adapted	66	10	201	83	360	0.00013	0.077045
C0304	101	V	I	NonAdapted	10	66	74	210	360	0.00052	0.179598
C0102	102	H	Y	NonAdapted	16	111	35	197	359	0.00012	0.071486
B1501	105	Q	K	NonAdapted	0	44	30	285	359	0.00025	0.114549
A0101	105	Q	K	Adapted	3	8	27	317	355	0.0005	0.167189
B3901	114	I	V	Adapted	6	16	35	298	355	0.00027	0.114549
B3901	114	V	V	NonAdapted	16	6	298	35	355	0.00027	0.114549
C0401	115	H	Y	Adapted	3	22	1	333	359	0.00054	0.17251
B5101	120	Y	Y	NonAdapted	13	39	177	130	359	2.96E-07	0.000611
B5101	120	F	Y	Adapted	35	17	127	180	359	4.95E-05	0.039204

C1403	120	F	Y	Adapted	22	10	140	187	359	0.0001	0.06247
C1403	120	Y	Y	NonAdapted	10	22	180	147	359	0.00014	0.080122
B4601	120	Y	Y	NonAdapted	14	23	176	146	359	0.00015	0.081455
B4601	120	F	Y	Adapted	22	15	140	182	359	0.00024	0.114117
C1402	125	Q	Q	NonAdapted	11	28	222	98	359	2.21E-08	5.48E-05
C1403	125	Q	Q	NonAdapted	10	22	218	104	354	1.02E-06	0.001807
A3303	125	H	Q	Adapted	13	24	50	271	358	0.00052	0.167189
B5101	125	H	Q	Adapted	20	32	43	264	359	0.00013	0.077045
C1403	125	H	Q	Adapted	13	19	50	277	359	0.00016	0.086431
C1402	126	G	N	Adapted	5	34	0	320	359	1.98E-06	0.002735
C1402	126	N	N	NonAdapted	25	14	296	24	359	4.35E-06	0.004909
B5401	126	N	N	NonAdapted	40	12	266	25	343	0.00023	0.110152
C1402	126	S	N	Adapted	7	32	9	311	359	0.00057	0.178568
B3501	133	I	T	NonAdapted	3	41	90	224	358	6.57E-05	0.054206
B3501	133	T	T	Adapted	34	10	149	165	358	0.00016	0.088014
A2402	135	Y	F	NonAdapted	21	177	104	56	358	5.40E-28	1.34E-23
A2402	135	F	F	Adapted	153	45	46	114	358	7.26E-20	9.01E-16
B3901	138	C	T	Adapted	5	17	24	312	358	0.00048	0.174964
A0206	152	D	Q	Adapted	3	56	1	283	343	0.00047	0.174964
C0602	157	N	N	Adapted	11	1	234	107	353	0.0002	0.103737
C0304	159	E	G	Adapted	6	68	0	280	354	1.35E-05	0.013534
B4006	161	D	N	Adapted	14	14	76	252	356	8.06E-05	0.057146
C1402	162	T	N	Adapted	6	33	6	311	356	0.0003	0.133582
B4006	162	N	N	NonAdapted	10	18	223	105	356	0.00039	0.160428
C0401	163	R	C	Adapted	2	23	12	319	356	0.00057	0.189021
A3303	174	E	D	NonAdapted	0	37	61	257	355	9.57E-05	0.066039
A3303	174	D	D	Adapted	37	0	257	61	355	9.59E-05	0.066039
B4002	174	D	D	Adapted	50	2	243	59	354	0.00012	0.071486
B4002	174	E	D	NonAdapted	2	50	59	243	354	0.00012	0.071486
A0207	174	E	D	Adapted	13	14	48	280	355	0.00052	0.179598
A0207	174	D	D	NonAdapted	14	13	280	48	355	0.00052	0.179598
A0207	177	D	E	Adapted	4	23	1	327	355	9.84E-05	0.066039
A0207	177	E	E	NonAdapted	23	4	327	1	355	9.84E-05	0.066039
A3101	188	R	R	NonAdapted	22	35	165	132	354	3.47E-06	0.004312
A3101	188	G	R	Adapted	4	53	1	296	354	0.00029	0.121041
A3101	192	R	H	NonAdapted	14	33	96	143	286	0.00014	0.081709
A3101	196	R	R	NonAdapted	40	17	273	24	354	1.36E-05	0.013534
A0207	201	E	E	NonAdapted	21	6	303	24	354	9.68E-05	0.066039
C0304	201	D	E	NonAdapted	0	75	19	260	354	0.00041	0.146607
C0702	201	Q	E	Adapted	3	101	7	242	353	0.00058	0.178568
A3101	207	R	*	Adapted	4	51	0	276	331	0.00015	0.082355

A

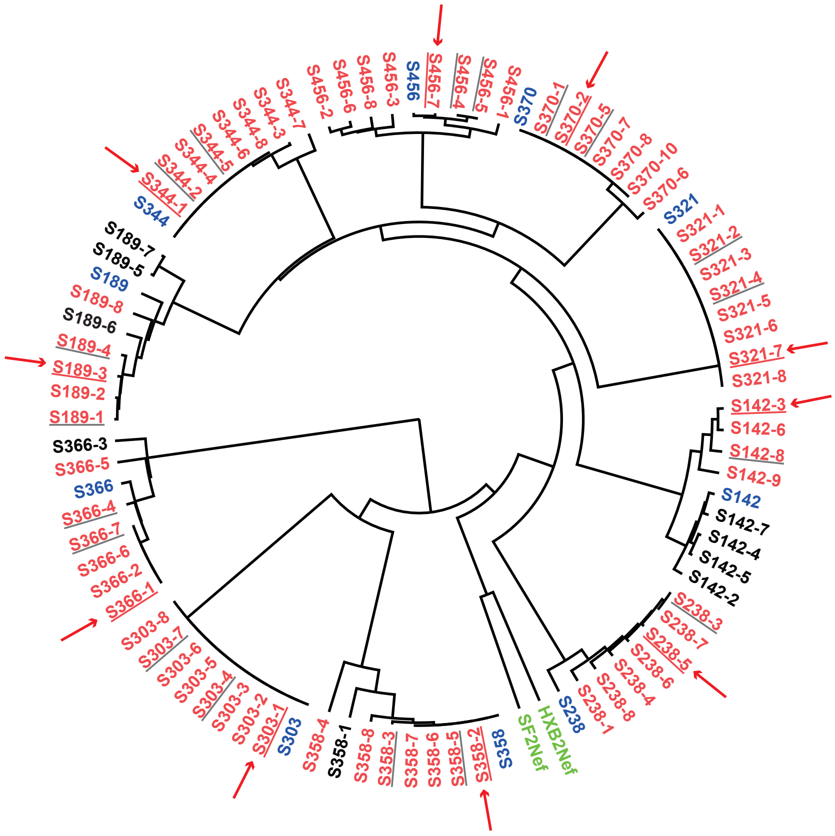

B

	120 125<br>TQGYFPDWQNYT	
S142	---F-----G--	3/8
	---FL----G--	1/8
	---F----H---	3/8
	---F----H-H-	1/8
S189	---F-----C--	3/8
	---F----H---	5/8
S238	---F----H---	7/7
S303	---F----H---	8/8
S321	---F----H---	8/8
S344	---F----H---	8/8
S358	---F--------	1/8
	---F----H---	7/8
S366	---F-----D--	1/7
	---F----H---	6/7
S370	---F----H---	7/7
S456	---F----H---	8/8

Fig. S1 Sequence analyses of nef clones isolated from plasma viral RNA of HIV-infected subjects
Maximum-likelihood phylogenetic tree *nef* sequences (panel A) and a part of Nef amino acid sequences of *nef* clones (panel B) isolated from plasma viral RNA of 10 HIV-infected patients (8 clones per patient) are shown. The phylogenetic tree of the *nef* sequences obtained from bulk PCR products and control strains of HXB2 and SF2 are shown in blue and green, respectively. The arrows indicate the Nef clones used for functional analyses (see text).

```
             1                                                              64
HXB2         MGGKWSKSSVIGWPTVRERMRRAEPAADRVGAASRDLEKHGAITSSNTAATNAACAWLEAQEEE
S142 clone3  ..S......IV...A.....K......EG...V...............N--H.D.V..K....D
S189 clone3  ..N....----.........K......EG...V..............VN--..D........D.
S238 clone5  .......C.--...A..K..EQ.....EG...................H--..D.V........
S303 clone1  .......R.IV...A.....N.T.....G...V....AR.........N--....V........
S321 clone7  ..S......IV...A.....K......EG...V...............N--..D.V......D.
S344 clone1  .........IV...A.....K.......G...V.............TAN--..D.V........
S358 clone2  ..S.V..--IV...A.....H......VG...V.....R..............D..........
S366 clone1  ..S......IV...A...K........EG...................N--..D.V........
S370 clone2  .........IV...A....IK......EG...V..............AN--..D..........
S456 clone7  .......T.IV...A.....Q......EG...V......R.......IN--..D.V..D...-.

65                                                                    135
EVGFPVTPQVPLRPMTYKAAVDLSHFLKEKGGLEGLIHSQRRQDILDLWIYHTQGYFPD-QNYTPGPGVRY
G.....R.........F.G.F......R.....D...Y....K......V.....F...WH.......T.L
......R.............F................Y..K.K......V.....F...WH..........
......K...........G.L...............VY..K.K......V.....F...WH.......T.W
......R..............................Y..K........V.....F...WH.......I..
......R.........F.G.L................Y..K.K......V.....F...WH.......T..
......R..............................Y....K......V.....F...WH.........L
......R..............................W..K..E.....V.....F...WH.......T.F
......R.........F.G.F......R........VY....K......V.....F...WH.......I..
......R...........G.F................Y..K.K......V.....F...WH.......T.F
......R.........F.G.L................Y..K.K......V.....F...WH.......I.F

136                                                                   206
PLTFGWCYKLVPVEPDKIEEANKGENTSLLHPVSLHGMDDPEREVLEWRFDSRLAFHHVARELHPEYFKNC
.......F.....D..QV.R..E..DN.....M.....E...G...M.K...H..LR.L...K...FYRD.
.......F.....D..QV.KE.E...N.....M.........K...M.K...L.....I...K...FY.D.
.......F.....D..QV.KE.E...N.....M.Q.......K...V.K...K.....M.K.K...FY.D.
.......F......KE.V....E...NC....M.Q...........M.K.........M.K.Q....Y.D.
.......F........QV.A..ER.D.N....M.........K...M.K...H.....M...K...FY.D.
.......F.....D..QV.KE.E...N.....M.....E...K...M.K...H..LR.L...K...FY.D.
.......F.......E.V...TV...NC....I.Q.......K...M.K.........M...K...FY.D.
.......F.....D..QV.KD.E..TNN....I.............V.K...S..L..R...K...FY.D.
.......F.....D..QV.KE.E..TNN....M.............M...........M...K...FY.D.
.......F.....D..QV.KE.E...N.....M....IE.......M.K.........M...K...FY.D.
```

Fig. S2 Amino acid sequence of the patient-derived Nef clones

Full-length amino acid sequences of the Nef clones selected for functional analyses (see Figures 4 and 5) are aligned in respect with the HXB2 strain. Dots denote amino acid residues identical to HXB2, and dashes denote the absence of amino acids at those positions.
